# Photodynamic therapy in chronic central serous chorioretinopathy with subretinal fluid outside the fovea

**DOI:** 10.1007/s00417-017-3720-z

**Published:** 2017-07-03

**Authors:** Elon H. C. van Dijk, Greet Dijkman, Camiel J. F. Boon

**Affiliations:** 1 Department of Ophthalmology, Leiden University Medical Center, Albinusdreef 2, 2333 ZA Leiden, The Netherlands; 2 Department of Ophthalmology, Academic Medical Center, University of Amsterdam, Amsterdam, The Netherlands

**Keywords:** Choroidal thickness, Chronic central serous chorioretinopathy, Extrafoveal, Photodynamic therapy, Subretinal fluid, Resolution, Visual complaints

## Abstract

**Purpose:**

To assess the efficacy of photodynamic therapy (PDT) in patients with chronic central serous chorioretinopathy (cCSC), in whom subretinal fluid (SRF) was solely present outside the foveal area.

**Methods:**

In this retrospective study, 16 eyes of 15 cCSC patients who received half-dose PDT because of notable subjective visual complaints due to the presence of extrafoveal SRF, were included. An ophthalmic examination was performed before treatment, including Early Treatment Diabetic Retinopathy Study best-corrected visual acuity measurement, applanation tonometry, slit-lamp examination, and indirect ophthalmoscopy, followed by multimodal imaging, including fundus photography, fundus autofluorescence, spectral-domain optical coherence tomography (OCT), enhanced-depth imaging OCT of the choroid, fluorescein angiography, and indocyanine green angiography.

**Results:**

In 7 treated patients (47%), PDT led to a decrease in visual complaints at the first evaluation visit. At this visit, extrafoveal SRF on OCT had resolved in 14 eyes (88%), whereas a complete resolution of extrafoveal SRF had occurred in all eyes at final follow-up visit. At baseline, posterior cystoid retinal degeneration was also present in 5 eyes (31%) and this remained present at all evaluation visits in these patients. Choroidal thickness decreased statistically significantly in the treated eyes, both foveally and at the location of the maximum height of extrafoveal SRF. No complications of PDT were observed.

**Conclusions:**

Half-dose PDT treatment of cCSC patients with visual complaints due to extrafoveal SRF accumulation is a safe procedure leading to complete SRF resolution, a decrease in choroidal thickness, and a reduction in visual symptoms.

**Electronic supplementary material:**

The online version of this article (doi:10.1007/s00417-017-3720-z) contains supplementary material, which is available to authorized users.

## Introduction

Central serous chorioretinopathy (CSC) is a chorioretinal disease that can eventually lead to vision loss as a result of irreversible retinal damage, mainly affecting middle-aged men. Despite the fact that the disease has already been described by Von Graefe in 1866, the exact pathogenetic mechanism of CSC is still unknown [1]. The disease is characterized by an accumulation of serous subretinal fluid (SRF). This leakage results from dysfunction of the retinal pigment epithelium (RPE) outer blood-retinal barrier, most probably caused by choroidal congestion, thickening, and hyperpermeability [2–5]. Compared to a control group, a significant increase in choroidal thickness (CT) in both affected eyes and non-affected fellow eyes has been described, supporting the hypothesis that CSC is a bilateral disorder which can present unilaterally [3, 6, 7].

In chronic CSC (cCSC), treatment is generally initiated in case of the presence of vision loss due to SRF accumulation under the fovea [4, 8]. Based on the currently available literature, photodynamic therapy (PDT) and micropulse laser treatment appear to be the most appropriate treatment modalities for the disease [8]. In up to 73% of cCSC patients, complete resolution of SRF can be achieved after micropulse laser treatment, whereas this occurs in up to 100% of cases after PDT [9–11]. However, no results of large randomized controlled treatment trials have been published yet [2].

PDT is thought to induce choroidal changes due to a temporary decrease in the perfusion of the choriocapillary layer and due to choroidal vascular remodeling, resulting in a reduction in fluid leakage from the choroid to the subretinal space [12]. However, some patients develop a temporary worsening of visual complaints in the first 2 weeks after PDT, and very rare complications such as choroidal ischemia, choroidal neovascularisation, and RPE atrophy have been described after PDT in cCSC [13–16]. Because of these possible complications and because of the fact that in cCSC patients extrafoveal SRF less often causes visual symptoms compared to foveal SRF, PDT is usually only performed in the cCSC patient group with foveal SRF. However, some patients without foveal SRF do have significant visual symptoms that require treatment. No studies on the use of PDT in cCSC patients with only extrafoveal SRF have been conducted thus far. In this retrospective study, we assessed the safety and efficacy of half-dose PDT on both visual complaints and SRF on optical coherence tomography (OCT) in cCSC patients with extrafoveal SRF.

## Methods

### Patients

Sixteen eyes of 15 patients could be included in this study. In these patients solely extrafoveal SRF could be detected on OCT prior to treatment. However, subjectively disabling visual complaints such as metamorphopsia, impaired color vision, and blurred peripheral vision led to the decision to schedule a treatment. PDT treatment had been performed between November 2014 and January 2017. Diagnosis of cCSC was established by fundoscopy, digital color fundus photography (Topcon Corp., Tokyo, Japan), fundus autofluorescence (Spectralis HRA + OCT; Heidelberg Engineering, Heidelberg, Germany), spectral-domain OCT (Spectralis HRA + OCT) and enhanced-depth imaging OCT of the choroid (Spectralis HRA + OCT), fluorescein angiography (FA; Spectralis HRA + OCT), and indocyanine green angiography (ICGA; Spectralis HRA + OCT). All of the following had to be present to set the cCSC diagnosis: disease duration of more than 4 months, serous SRF on OCT, ≥ one area of a ‘hot spot’ of leakage or diffuse leakage in combination with irregular RPE window defects on FA, and corresponding hyperfluorescence on ICGA. Patients in whom evidence of other diagnoses than cCSC were present, or cases with evidence of complications such as polypoidal choroidal vasculopathy and/or choroidal neovascularisation, were excluded.

Local ethics committee and institutional review board approval was obtained. The study followed the tenets of the Declaration of Helsinki.

### Photodynamic therapy treatment

Half-dose intravenous (3 mg/m^2^) verteporfin (Visudyne®; Novartis Europharm Ltd., Horsham, West Sussex, UK) was administrated over a period of 10 min. At exactly 15 min after the start of the verteporfin infusion, a contact glass (Volk® PDT lens) was positioned on the affected eye, and the laser beam was projected on the area to be treated. The zone to be treated was chosen based on hyperfluorescent areas on mid-phase (10′) ICGA, corresponding to SRF on OCT and hyperfluorescent ‘hot spots’ of leakage on mid-phase (3′) FA. For the PDT treatment, a fluency of 50 J/cm^2^, treatment duration of 83 s, and a laser wavelength of 689 nm (Carl Zeiss Meditec, Dublin, CA, USA) were used.

### Ophthalmological examinations

Ocular complaints were recorded and Early Treatment Diabetic Retinopathy Study (ETDRS) best-corrected visual acuity (BCVA) was measured at the last visit before PDT and at least at one evaluation visit after PDT. When ETDRS BCVA was not available, a previously described conversion method was used [17]. The effect of treatment on SRF was assessed with spectral-domain OCT imaging. Moreover, the effect on intraretinal cystoid spaces without intraretinal leakage (posterior cystoid retinal degeneration) was also studied on these OCT images [18, 19].

For the treated eyes, the following findings were measured manually on enhanced-depth imaging (EDI)-OCT with use of the caliper tool in Heidelberg Eye Explorer (version 1.9.10.0; Heidelberg Engineering) at the last visit before PDT and at least at one evaluation visit after PDT: foveal CT (distance from the outer part of the hyperreflective RPE layer to the hyperreflective line of the inner surface of the sclera, on EDI-OCT) and CT at the location of the maximum height of extrafoveal SRF (distance from the outer part of the external limiting membrane to the outer part of the RPE layer). Complete resolution of SRF on OCT was considered to be the desired anatomical treatment effect. For comparison with CT in the treated eyes, subfoveal CT was also measured on EDI-OCT in untreated fellow eyes.

### Statistical analysis

For statistical analyses, a dependent *t* test was used in SPSS Statistics (version 23; IBM Corp., Armonk, NY, USA) to compare both ETDRS and CT at evaluation visits with ETDRS and CT before PDT. The level of statistical significance was set at *p* < 0.05.

## Results

The 15 cCSC patients (16 eyes; 14 male patients, 1 female patient) who were included in this study had a mean age of 52 ± 13 years (range, 35–80 years). In the treated eyes, cCSC had been diagnosed for the first time at 21 ± 21 months (range, 3–83 months) before PDT. Prior to PDT treatment, ETDRS BCVA in the affected eyes was 78 (Snellen equivalent: 20/29) ± 18 letters (range, 21–95 letters). Bilateral signs of cCSC were present in 10 patients (67%). Posterior cystoid retinal degeneration was present in 5 eyes (31%) at the last visit before PDT. Nine included eyes (56%) had received previous CSC treatment because of foveal SRF, including micropulse laser treatment (7 eyes) and half-dose PDT (2 eyes). All patients had received this previous treatment within 1 year before the half-dose PDT performed within this study. The mean PDT spot size in the treated eyes was 5.0 ± 1.7 mm (range, 2.2–7.2 mm), and the verteporfin dosage was 2.9 ± 0.4 ml (range, 2.0–3.3 ml). Two patients were treated with two PDT spots in one session. Out of 12 treated eyes of 12 patients, for which this information was available, the fovea was included in the treatment spot in 10 eyes (84%). Patient characteristics are summarized in Table 1.

**Table 1:** Clinical characteristics of patients with central serous chorioretinopathy who received photodynamic therapy for extrafoveal subretinal fluid

Patient	Age	Gender	Baseline ETDRS BCVA	Duration of CSC (days)	Bilateral CSC	Recurrent CSC	Previous CSC treatment-affected eye
1	50	M	85	352	Y	N	N
2	51	F	86	488	N	N	Micropulse laser treatment (2*)
3	70	M	77	421	Y	Y	N
4	37	M	59	1299	Y	Y	N
5	80	M	21	129	Y	N	Half-dose photodynamic therapy (foveal SRF)
6	72	M	89	725	Y	N	N
7	35	M	72	474	Y	Y	Micropulse laser treatment (1*)
8	51	M	90	547	N	Y	Micropulse laser treatment (2*)
9	47	M	92	1518	Y	N	Half-dose photodynamic therapy (foveal SRF)
10	49	M	84	333	N	N	Micropulse laser treatment (2*)
11	44	M	90	349	N	N	Micropulse laser treatment (2*)
12	41	M	67	315	Y	N	Micropulse laser treatment (2*)
13	50	M	76	671	N	N	Micropulse laser treatment (2*)
14	47	M	95	211	Y	N	N
15	57	M	81	83	Y	N	N
	57	M	75	2523	NA	Y	N

BCVA = best-corrected visual acuity, CSC = central serous chorioretinopathy, ETDRS = Early Treatment of Diabetic Retinopathy Study, NA = not applicable, SRF = subretinal fluid.

At the first evaluation visit after a mean of 63 days (range, 7–161 days) after half-dose PDT, a reduction in visual symptoms had occurred in 7 patients (47%). At that visit, ETDRS BCVA was 80 (Snellen equivalent: 20/25) ± 16 letters (range, 35–96 letters), which was not statistically significantly different from the last visit before PDT (*p* = 0.074). At the last evaluation visit after a mean of 325 days (range, 189–351 days) after PDT, ETDRS BCVA was 78 (Snellen equivalent: 20/29) ± 20 letters (range, 20–93 letters) as compared to the last visit before PDT (*p* = 0.836).

**Fig. 1:**
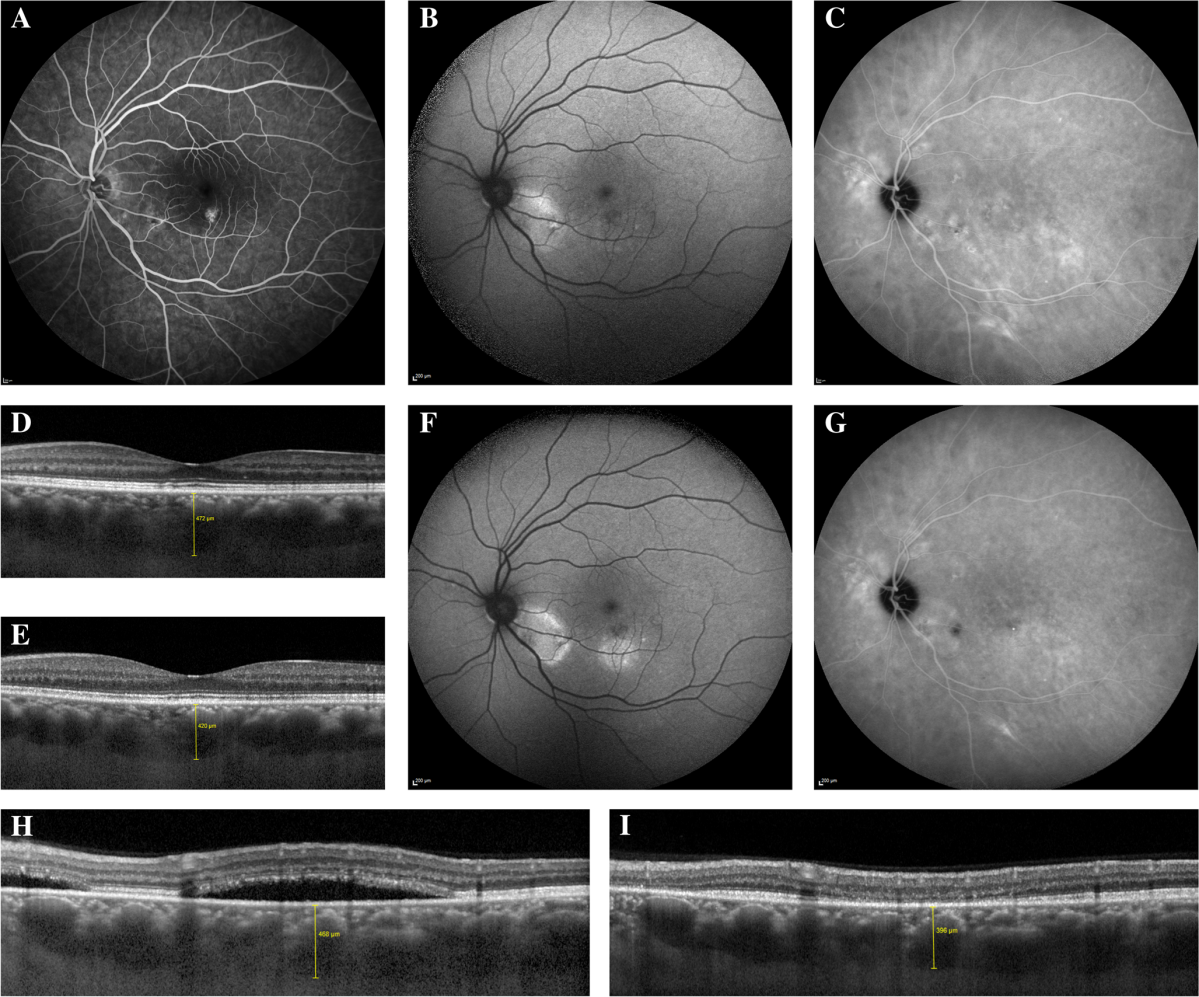
Characteristics on multimodal imaging of a patient with chronic central serous chorioretinopathy with only extrafoveal subretinal fluid, who was treated with half-dose photodynamic therapy. (**A–D**) Fluorescein angiography (FA; A), fundus autofluorescence (FAF) imaging (B), indocyanine green angiography (ICGA; C), and a foveal optical coherence tomography (OCT) scan (D) of a 47-year-old male patient with chronic central serous chorioretinopathy before half-dose photodynamic therapy (PDT). FA showed hyperfluorescent areas, with a ‘hot spot’ of leakage inferiorly of the fovea. On FAF, both hyper- and hypoautofluorescent abnormalities were present. ICGA revealed the presence of areas of hyperfluorescence, larger compared to the extent of the abnormalities on FA. On the foveal OCT scan, no subretinal fluid (SRF) was present. Some subtle retinal pigment epithelium abnormalities could be detected, and the choroidal thickness (CT) was 472 μm. (**E–G**) At the evaluation visit at 6 weeks after half-dose PDT, the subfoveal CT on OCT (E) had decreased to 420 μm. FAF imaging (F) revealed a slight increase in the extent of hyperautofluorescent abnormalities, but the extent of hyperfluorescent abnormalities on ICGA (G) had clearly decreased. (**H–I**) On the OCT scan (H), inferiorly of the fovea, obtained before half-dose PDT, on which the maximum height of extrafoveal SRF could be detected, the CT was 468 μm. At 6 weeks after treatment, a complete resolution of SRF on OCT (I) had occurred and the CT had decreased to 396 μm. The complaints of the patient, which mainly consisted of metamorphopsia, had not changed at the evaluation visit after treatment

A complete resolution of extrafoveal SRF had occurred in 14 eyes (88%) at the first evaluation visit after PDT, whereas posterior cystoid retinal degeneration had not disappeared in any of the eyes. In the 9 patients for whom EDI-OCT images of sufficient quality were available both before PDT and at the first evaluation, the subfoveal CT was 408 *±* 93 μm (range, 200–509 μm) at this first visit after PDT, which was a significant decrease as compared to before PDT [452 *±* 95 μm (range, 238–554 μm); *p* = 0.043]. CT at the location of the maximum height of extrafoveal SRF was 407 *±* 99 μm (range, 213–544 μm) before PDT, which was statistically significantly higher compared to CT at the first evaluation after PDT [351 *±* 102 μm (range, 172–489 μm); *p* = 0.015]. Before PDT, subfoveal CT in the untreated fellow eye was 415 *±* 91 μm (range, 244–489 μm), which did not differ from CT at the first evaluation visit [411 *±* 96 μm (range, 224–502 μm); *p* = 0.711]. At the final follow-up visit of 6 patients for whom EDI-OCT images of sufficient quality were available, the subfoveal CT was 397 *±* 50 μm (range, 341–468 μm) and the extrafoveal CT was 371 *±* 84 μm (range, 245–475 μm), which was significantly lower than the CT before PDT (*p* = 0.001 and *p* = 0.﻿003, respectively). At that moment, the CT of the untreated fellow eyes was 422 *±* 78 μm (range, 303–496 μm), which did not differ from CT before PDT (*p* = 0.953). Multimodal imaging of a patient before and after half-dose PDT is depicted in Fig. 1.

At final evaluation visit, extrafoveal SRF had disappeared in all patients. The two patients in whom extrafoveal SRF had not resolved at the first evaluation visit received an additional half-dose PDT treatment, after which a complete resolution of SRF occurred. Both patients had previously received treatment for cCSC, including micropulse laser treatment (one eye) and half-dose PDT (one eye). Characteristics on OCT before and at the evaluation visits after half-dose PDT are summarized in Table 2.

**Table 2:** Characteristics on optical coherence tomography in chronic central serous chorioretinopathy patients who received half-dose photodynamic therapy for extrafoveal subretinal fluid

Patient	Reduction of complaints after PDT	Pre-PDT SCT (μm)	Post1-PDT SCT (μm)	Post2-PDT SCT (μm)	Pre-PDT extrafoveal CT (μm)	Post1-PDT extrafoveal CT (μm)	Post2-PDT extrafoveal CT (μm)	Pre-PDT SCT fellow eye (μm)	Post1-PDT SCT fellow eye (μm)	Post2-PDT SCT fellow eye (μm)
1	Y	NA	NA	NA	NA	NA	NA	NA	NA	NA
2	Y	549	420	430	482	385	365	478	447	496
3	N	NA	NA	NA	NA	NA	NA	NA	NA	NA
4	N	NA	NA	NA	NA	NA	NA	NA	NA	NA
5	N	NA	NA	NA	NA	NA	NA	NA	NA	NA
6	N	NA	NA	NA	NA	NA	NA	NA	NA	NA
7	Y	NA	NA	NA	NA	NA	NA	NA	NA	NA
8	N	NA	NA	NA	NA	NA	NA	NA	NA	NA
9	N	413	427	NA	334	348	NA	489	416	NA
10	N	238	200	NA	344	268	NA	244	224	NA
11	Y	485	372	341	441	294	245	472	475	451
12	Y	416	361	344	382	324	306	348	368	354
13	Y	447	482	NA	213	172	NA	465	469	NA
14	N	492	486	385	454	485	410	458	496	492
15	Y	554	509	468	544	489	475	475	502	437
	Y	472	420	413	468	396	423	306	299	303

CT = choroidal thickness, NA = not available, PDT = photodynamic therapy, Post1 = first evaluation visit after PDT, Post2 = last evaluation visit after PDT, SCT = subfoveal choroidal thickness.

## Discussion

To the best of our knowledge, this is the first study describing the outcome of PDT treatment in cCSC patients in whom only extrafoveal SRF was present on OCT. A complete resolution of SRF occurred in 88% of patients at first evaluation visit, and in all patients at final follow-up visit. Also, CT in the treated eyes decreased significantly at the evaluation visits both at the location of the maximum height of extrafoveal SRF and in the fovea. A decrease in visual complaints was reported for 47% of treated patients.

The percentage of patients in whom SRF disappeared after half-dose PDT is in line with the outcome of other studies that included patients with foveal SRF, for whom it has been described that treatment could prevent the occurrence of permanent photoreceptor damage [9, 20, 21]. This complete resolution occurred despite the fact that the majority of eyes in our study had previously received treatment for cCSC. In addition to a resolution of SRF, treatment resulted in a significant reduction in CT, both extrafoveally and foveally. A comparable subfoveal CT reduction independently from including the fovea in the PDT-treated area has been previously described [11, 22]. Such an effect that is distant from the area that was actually treated with the PDT spot may be explained by choroidal remodeling after PDT treatment [12]. Apart from the finding that SRF and visual symptoms resolved in a noteworthy number of cCSC patients with extrafoveal SRF included in this study, these treatment effects in these cCSC patients may also decrease the likelihood of either recurrence of SRF or progression to foveal SRF leakage at a later date, which could lead to irreversible damage [23].

Based on the available, mostly retrospective evidence on the safety and efficacy of PDT using reduced treatment settings in cCSC [8, 13], our first-line choice for the treatment of cCSC is PDT, and this treatment resulted in a complete resolution of SRF in all the included patients with extrafoveal SRF. However, the optimal treatment and timing of treatment for cCSC is subject to controversy, due to the lack of large prospective randomized controlled trials. We are currently performing a large prospective randomized controlled multicenter treatment trial, the PLACE trial, comparing half-dose PDT with high-density subthreshold micropulse laser treatment for cCSC [24]. In this trial, both anatomical and functional parameters are taken into account, for a prolonged follow-up period [24]. However, for patients included in this trial and in other treatment trials on cCSC, generally, the presence of SRF in the fovea is mandatory to be eligible for inclusion. Limitations of the current study include the small number of included patients, its retrospective nature, and the relatively short follow-up period. Since PDT treatment can lead to both a temporary increase in visual complaints and to serious complications, it should be performed with its associated risks in mind, especially in patients without SRF in the fovea [13–16].

In conclusion, half-dose PDT treatment of cCSC patients with notable visual complaints due to extrafoveal SRF accumulation induces complete SRF resolution and leads to a decrease in CT and a reduction in visual symptoms.

## Electronic supplementary material

ESM 1 (XLSX 5501 kb).
